# Supplementary material for: Gastrointestinal Permeation Enhancers for the Development of Oral Peptide Pharmaceuticals
Source: Pharmaceuticals (Basel). 2022 Dec 19;15(12):1585. doi: 10.3390/ph15121585 (PMC9781085; doi:10.3390/ph15121585)
Supplement: Supplementary file 1 [file pharmaceuticals-15-01585-s001.zip › pharmaceuticals-2050550-supplementary.pdf]

Supplementary Materials

Gastrointestinal Permeation Enhancers for the Development of Oral Peptide Pharmaceuticals

Jae Cheon Kim [1], Eun Ji Park [2,*] and Dong Hee Na [1,*]

[1] College of Pharmacy, Chung-Ang University, Seoul 06974, Republic of Korea
[2] D&D Pharmatech, Seongnam, Gyeonggi-do 13486, Republic of Korea
* Correspondence: ejpark@ddpharmatech.com (E.J.P.); dhna@cau.ac.kr (D.H.N)

Table S1. Clinical studies of oral drug formulations using sodium caprate (C10).

Table S2. Clinical studies of oral drug formulations using SNAC.

Table S1. Clinical studies of oral drug formulations using sodium caprate (C10).

Drug	Sponsor/ Collaborator	Description	Outcome	Clinical Trials (NCT code/Study completion year/Publication)
Insulin 338	Novo Nordisk A/S	Insulin 338 with C10 (GIPET™ 1) in subjects with Type 2 DM (n = 40) (To investigate the safety, tolerability, PK and PD)	disclosed	Phase 1 (NCT01796366/2013)
Insulin 338	Novo Nordisk A/S	Insulin 338 with C10 (GIPET™ 1) in healthy subjects (n = 40) (To investigate the PK/PD in a tablet formulation with three different coatings)	disclosed	Phase 1 (NCT01931137/2013)
Insulin 338	Novo Nordisk A/S	Insulin 338 with C10 (GIPET™ 1) in healthy subjects (n = 45) (To investigate the effect of food on the PK)	Oral insulin 338 PK are not affected by food intake from 30 min after dosing.	Phase 1 (NCT02304627/2015) [1]
Insulin 338	Novo Nordisk A/S	Insulin 338 with C10 (GIPET™ 1) in subjects with Type 2 DM (n = 50) over 8 weeks. (To compare insulin-338 and insulin glargine)	Insulin 338 can safely improve glycemic control with no evidence of a difference compared with insulin glargine. However, it is not considered commercially viable due to its low BA (1.5-2.0 % compared to insulin glargine)	Phase 2 (NCT02470039 / 2015) [2]

Insulin 287	Novo Nordisk A/S	Insulin 287 with C10 (GIPET™ 1) in healthy subjects (n = 84) (To investigate the safety, tolerability, PK and PD)	disclosed	Phase 1 (NCT01809184 / 2013)
Acyline	University of Washington /Merrion Pharmaceuticals	GIPET™ enhanced oral acyline (MER-104) in normal men (n = 9) (To investigate the safety, efficacy, PK and PD)	Significant reduction in serum LH, FSH, and Testosterone were observed after 6 hours at all concentrations (10, 20, and 40 mg of acyline)	Phase 1 (NCT00471185 / 2007) [3]
Acyline	University of Washington /Merrion Pharmaceuticals	GIPET™ enhanced oral acyline (MER-104-02) in normal men (n = 4) (To determine steady-state (multiple-dese) PK)	Has a result	Phase 1/2 (NCT00603187 / 2011)
Insulin Tregopil	Biocon	Insulin Tregopil with C10 as PE in healthy subjects (n = 48) (To evaluate the effect of insulin tregopil and C10 on PK of metformin under fed and fasting conditions.)	C10/insulin tregopil did not affect the PK of metformin in either the fasting/fed state.	Phase 1 (2014) [4]

Table S2. Clinical studies of oral drug formulations using SNAC.

Drug	Sponsor/ Collaborator	Description	Outcome	Clinical Trials (NCT code/Study completion year/Publication)
		SNAC		
Vitamin B12	Emisphere technologies	Vitamin B12 with SNAC in healthy subjects (n = 20) (To assess the PK of vitamin B12)	5 mg of vitamin B12 with 100 mg of SNAC was selected, indicating a BA of 5.09%. (2.16% for commercial products)	Phase Not applicable (NCT01311739/2008) [5]
Unfractionated heparin	Emisphere technologies	Unfractionated heparin with SNAC in healthy subjects (n = 16) (To determine the PK/PD profile of oral unfractionated heparin)	Oral heparin alone did not show absorption, and heparin/SNAC combined administration showed that the BA based on anti-factor Xa and anti-factor II was 0.0219 and 0.0133%, respectively	[6]
Insulin	Emisphere technologies	Insulin with SNAC in non-diabetic subjects (n = 16) (To examine the absorption of insulin from the GI tract, using SNAC)	In all cases, a hypoglycemic effect was observed, and an increase in plasma insulin level was observed	[7]
GLP-1 and PYY3-36	University Hospital, Basel, Switzerland/ Emisphere technologies	GLP-1, PYY3-36 with SNAC in healthy male subjects (n = 16) (To explore potential interactions between two satiety signals)	Experiments on the enhancement of oral peptide absorption by SNAC are not designed	Phase 1 / 2 (NCT00822705 / 2009) [8]

Semaglutide	Novo Nordisk A/S	Semaglutide/SNAC formulation with three different dosing conditions in healthy subjects (n = 78) (To investigate the effect of food on the PK of oral semaglutide)	Limited or no measurable semaglutide exposure was observed in the fed state. Measurable semaglutide exposure was observed in the fasting state, and the AUC was also about 40% greater.	Phase 1 (NCT02172313 / 2014) [9]
Semaglutide	Novo Nordisk A/S	Semaglutide with SNAC in subject with renal impairment and with normal renal function (n = 71) (To investigate the PK, safety and tolerability of oral semaglutide in subjects with various degree of impaired renal function)	There was no apparent effect of renal impairment or haemodialysis on the PK of oral semaglutide.	Phase 1 (NCT02014259 / 2014) [10]
Semaglutide	Novo Nordisk A/S	Semaglutide with SNAC in subject with hepatic impairment and with normal hepatic function (n = 56) (To investigate the PK, safety and tolerability of oral semaglutide in subjects with mild, moderate and severe degrees of hepatic impairment)	The PK parameters were similar across hepatic function groups. No safety concern were identified in subjects with hepatic impairment receiving oral semaglutide.	Phase 1 (NCT02016911 / 2015) [11]
Semaglutide	Novo Nordisk A/S	Semaglutide with SNAC in subject with T2DM with or without upper GI disease (n = 55) (To investigate the effect of upper GI disease on the PK of oral semaglutide)	The modelled PK profile were similar for subject with or without upper GI disease	Phase 1 (NCT02877355 / 2017) [12]
Semaglutide	Novo Nordisk A/S	Semaglutide with SNAC in subject with T2DM (n = 632) (To examine the dose range, escalatioin and efficacy of oral semaglutide)	Oral semaglutide from 2.5 mg to 40 mg decreased HbA_{1c} from 0.7% to 1.9% in a dose-dependent manner. The most common adverse	Phase 2 (NCT01923181 / 2014) [13]

			event was mild to moderate gastrointestinal events.	
Semaglutide	Novo Nordisk A/S	Semaglutide with SNAC in subject with T2DM treated with diet and exercise only (n = 703) (To investigate efficacy and safety of oral semaglutide)	Oral semaglutide at 3 mg, 7 mg, and 14 mg combined with SNAC resulted in HbA_{1c} reduction of 0.7%,1.2%, and 1.4%, respectively, and the 14 mg dose resulted in a significant weight loss of 2.3 kg	Phase 3 (NCT02906930 / 2017) [14]
Semaglutide	Novo Nordisk A/S	Semaglutide with SNAC in subject with T2DM uncontrolled on metformin (n = 822) (To investigate efficacy and safety of oral semaglutide versus empagliflozin)	Oral semaglutide showed a better HbA_{1c} reduction compared to emphagliflozin in the group administered for 26 weeks (-1.3% vs -0.9%), and the group administered for 52 weeks showed a better weight loss.	Phase 3 (NCT02863328 / 2019) [15]
Semaglutide	Novo Nordisk A/S	Semaglutide with SNAC in subject with T2DM and moderate renal impairment (n = 324) (To investigate efficacy and safety of oral semaglutide versus placebo)	When administered for 26 weeks, the estimated treatment differences were 0.8% and 2.5 kg, respectively, compared to placebo in HbA_{1c} reduction and weight loss. More adverse events occurred than in the placebo group, and most were mild-to-moderate GI events.	Phase 3 (NCT02827708 / 2017) [16]

Semaglutide	Novo Nordisk A/S	Semaglutide/SNAC alone or Semaglutide/SNAC with omeprazole in healthy subjects (n = 54)<br>(To investigate the influence of omeprazole on the PK of oral semaglutide)	There was a slight non-statistically significant increase in semaglutide exposure when oral semaglutide was administered with omeprazole, but this is not considered clinically relevant and no dose adjustment is likely to be required.	Phase 1<br>(NCT02249871 / 2015)<br>[17]
Semaglutide	Novo Nordisk A/S	Lisinopril or warfarin with subsequent coadministration with SNAC alone followed by oral semaglutide in healthy subjects (n = 52)<br>(To investigate the influence of oral semaglutide on the PK of lisinopril and warfarin)	There was no apparent effect of oral semaglutide on AUC and C_{max} for lisinopril and warfarin.	Phase 1<br>(NCT02070510 / 2014)<br>[18]
Semaglutide	Novo Nordisk A/S	Digoxin or metformin with subsequent coadministration with SNAC alone followed by oral semaglutide in healthy subjects (n = 32)<br>(To investigate the influence of oral semaglutide on PK of metformin and digoxin)	There was no apparent effect of oral semaglutide on AUC and C_{max} for digoxin.<br>The AUC of metformin was increased by 32% by oral semaglutide coadministration versus metformin alone, whereas the C_{max} was unaffected.	Phase 1<br>(NCT02249910 / 2015)<br>[18]
Semaglutide	Novo Nordisk A/S	Ethinylestradiol/Levonogestrel alone or together with oral semaglutide or SNAC in healthy postmenopausal females (n = 25)<br>(To investigate the influence of oral semaglutide on PK of ethinylestradiol and levonogestrel)	The AUC and C_{max} of ethinylestradiol and levonogestrel were not affected by oral semaglutide and SNAC administration	Phase 1<br>(NCT02845219 / 2016)<br>[19]

SNAC	Novo Nordisk A/S	SNAC in healthy male subjects (n = 84) (To evaluate the effect of SNAC on cardiac repolarization)	There was no unacceptable prolongation of the QTc interval with SNAC 3.6 g, the maximum investigated SNAC	Phase 1 (NCT02911870 / 2017) [20]
Semaglutide	Novo Nordisk A/S	Levothyroxine alone, or with concomitant SNAC or concomitant oral semaglutide in healthy subjects (n = 45) (To investigate the influence of oral semaglutide on the PK of levothyroxine and the influence of co-administered tablets on the PK of semaglutide)	A 33% increase in total T4 exposure was observed with levothyroxine/oral semaglutide vs levothyroxine alone. A 34% decrease in semaglutide AUC was observed when oral semaglutide was co-administered with placebo tablet.	Phase 1 (NCT02920385 / 2017) [21]
Semaglutide	Novo Nordisk A/S	Single doses of furosemide and rosuvastatin alone, with SNAC alone or with oral semaglutide in healthy subjects (n = 41) (To investigate the effect of oral semaglutide on the PK of furosemide and rosuvastatin)	The no-effect-criterion was not met for furosemide and rosuvastatin for the AUC (estimated ratios [90% CI] 1.28[1.16-1.42] and 1.41[1.24-1.60], respectively). SNAC alone did not affect the AUC or C_{max} of rosuvastatin; the C_{max} of furosemide was slightly decreased.	Phase 1 (NCT03010475 / 2017) [19]
Semaglutide	Novo Nordisk A/S	Semaglutide /SNAC alone, ciclosporin with semaglutide /SNAC, and probenecid with semaglutide/SNAC in healthy subjects (n = 21) (To investigate the effect of the medicines, probenecid and ciclosporin on the concentration of SNAC)	Disclosed	Phase 1 (NCT03466567 / 2018)

Semaglutide	Novo Nordisk A/S	Semaglutide with SNAC in healthy, lactating females (n = 14) (To investigate how much semaglutide and SNAC is in the milk)	-	Phase 1 (NCtT04817644 / Recruiting)
Semaglutide	Novo Nordisk A/S	Semaglutide with SNAC in children and teenagers with T2DM (n = 132) (To test if semaglutide is safe in children and teenagers)	-	Phase 3 (NCtT04596631 / Recruiting)
Semaglutide	Novo Nordisk A/S	Current semaglutide/SNAC formulation (includes 3 helping agents) or new semaglutide/SNAC formulation (includes only one helping agent) in healthy subjects (n = 278) (To find out if the dosage strength of the current formulation of semaglutide can be reduced in the new formulation)	Disclosed	Phase 1 (NCT04109508 / 2021)
Semaglutide	Novo Nordisk A/S	Current semaglutide/SNAC formulation or 4 new semaglutide/SNAC formulations with different helping agents in different amounts in healthy subjects (n = 290) (To test oral semaglutide of 25 mg and 50 mg)	Disclosed	Phase 1 (NCT04524832 / 2022)
NNC0113-2023 (GLP-1R agonist)	Novo Nordisk A/S	NNC0113-2023 with SNAC in healthy male subjects (n = 40) (To assess safety and participant tolerability, To measure the concentration	Disclosed	Phase 1 (NCT03617081 / 2018)

		and of NNC0113-2023 and its breakdown products)		
NNC0385-0434 (PCSK9 inhibitor)	Novo Nordisk A/S	NNC0385-0434 with SNAC in healthy subjects (n = 73) (To investigate the effect of food intake on the amount of NNC0385-0434 in the blood)	Disclosed	Phase 1 (NCT05091073 / 2022)
NNC0385-0434 (PCSK9 inhibitor)	Novo Nordisk A/S	NNC0385-0434 with SNAC in healthy japanese male (n = 36) (To look at how NNC0385-0434 works in the body and how it is removed from the body)	Disclosed	Phase 1 (NCT05003440 / 2022)
NNC0385-0434 (PCSK9 inhibitor)	Novo Nordisk A/S	NNC0385-0434 with SNAC in subjects with established ASCVD or ASCVD risk on maximally tolerated statin dose and other lipid-lowering therapy requiring further LDL-C reduction (n = 255) (To investigate efficacy)	Disclosed	Phase 2 (NCT04992065 / 2022)
NNC0385-0434 (PCSK9 inhibitor)	Novo Nordisk A/S	NNC0385-0434 with SNAC in healthy subjects and subjects with impaired kidney function (n = 60) (To investigate of PK, safety and tolerability of oral NNC0385-0434 in subjects with various degree of impaired	-	Phase 1 (NCT05094934 / Recruiting)

	renal function and in subjects with normal renal function)	

References

1. Halberg, I.B.; Lyby, K.; Wassermann, K.; Heise, T.; Plum-Mörschel, L.; Zijlstra, E. The Effect of Food Intake on the Pharmacokinetics of Oral Basal Insulin: A Randomised Crossover Trial in Healthy Male Subjects. *Clin Pharmacokinet* **2019**, *58*, 1497-1504, doi:10.1007/s40262-019-00772-2.
2. Halberg, I.B.; Lyby, K.; Wassermann, K.; Heise, T.; Zijlstra, E.; Plum-Mörschel, L. Efficacy and safety of oral basal insulin versus subcutaneous insulin glargine in type 2 diabetes: a randomised, double-blind, phase 2 trial. *Lancet Diabetes Endocrinol* **2019**, *7*, 179-188, doi:10.1016/s2213-8587(18)30372-3.
3. Amory, J.K.; Leonard, T.W.; Page, S.T.; O'Toole, E.; McKenna, M.J.; Bremner, W.J. Oral administration of the GnRH antagonist acyline, in a GIPET-enhanced tablet form, acutely suppresses serum testosterone in normal men: single-dose pharmacokinetics and pharmacodynamics. *Cancer Chemother Pharmacol* **2009**, *64*, 641-645, doi:10.1007/s00280-009-1038-1.
4. Khedkar, A.; Lebovitz, H.; Fleming, A.; Cherrington, A.; Jose, V.; Athalye, S.N.; Vishweswaramurthy, A. Impact of Insulin Tregopil and Its Permeation Enhancer on Pharmacokinetics of Metformin in Healthy Volunteers: Randomized, Open-Label, Placebo-Controlled, Crossover Study. *Clin Transl Sci* **2019**, *12*, 276-282, doi:10.1111/cts.12609.
5. Castelli, M.C.; Wong, D.F.; Friedman, K.; Riley, M.G. Pharmacokinetics of oral cyanocobalamin formulated with sodium N-[8-(2-hydroxybenzoyl)amino]caprylate (SNAC): an open-label, randomized, single-dose, parallel-group study in healthy male subjects. *Clin Ther* **2011**, *33*, 934-945, doi:10.1016/j.clinthera.2011.05.088.
6. Mousa, S.A.; Zhang, F.; Aljada, A.; Chaturvedi, S.; Takieddin, M.; Zhang, H.; Chi, L.; Castelli, M.C.; Friedman, K.; Goldberg, M.M.; et al. Pharmacokinetics and pharmacodynamics of oral heparin solid dosage form in healthy human subjects. *J Clin Pharmacol* **2007**, *47*, 1508-1520, doi:10.1177/0091270007307242.
7. Kidron, M.; Dinh, S.; Menachem, Y.; Abbas, R.; Variano, B.; Goldberg, M.; Arbit, E.; Bar-On, H. A novel per-oral insulin formulation: proof of concept study in non-diabetic subjects. *Diabet Med* **2004**, *21*, 354-357, doi:10.1111/j.1464-5491.2004.01160.x.
8. Steinert, R.E.; Poller, B.; Castelli, M.C.; Drewe, J.; Beglinger, C. Oral administration of glucagon-like peptide 1 or peptide YY 3-36 affects food intake in healthy male subjects. *Am J Clin Nutr* **2010**, *92*, 810-817, doi:10.3945/ajcn.2010.29663.
9. Bækdal, T.A.; Breitschaft, A.; Donsmark, M.; Maarbjerg, S.J.; Søndergaard, F.L.; Borregaard, J. Effect of Various Dosing Conditions on the Pharmacokinetics of Oral Semaglutide, a Human Glucagon-Like Peptide-1 Analogue in a Tablet Formulation. *Diabetes Ther* **2021**, *12*, 1915-1927, doi:10.1007/s13300-021-01078-y.
10. Granhall, C.; Søndergaard, F.L.; Thomsen, M.; Anderson, T.W. Pharmacokinetics, Safety and Tolerability of Oral Semaglutide in Subjects with Renal Impairment. *Clin Pharmacokinet* **2018**, *57*, 1571-1580, doi:10.1007/s40262-018-0649-2.
11. Baekdal, T.A.; Thomsen, M.; Kupčová, V.; Hansen, C.W.; Anderson, T.W. Pharmacokinetics, Safety, and Tolerability of Oral Semaglutide in Subjects With Hepatic Impairment. *J Clin Pharmacol* **2018**, *58*, 1314-1323, doi:10.1002/jcph.1131.
12. Overgaard, R.V.; Navarria, A.; Ingwersen, S.H.; Bækdal, T.A.; Kildemoes, R.J. Clinical Pharmacokinetics of Oral Semaglutide: Analyses of Data from Clinical Pharmacology Trials. *Clin Pharmacokinet* **2021**, *60*, 1335-1348, doi:10.1007/s40262-021-01025-x.
13. Davies, M.; Pieber, T.R.; Hartoft-Nielsen, M.L.; Hansen, O.K.H.; Jabbour, S.; Rosenstock, J. Effect of Oral Semaglutide Compared With Placebo and Subcutaneous Semaglutide on Glycemic Control in Patients With Type 2 Diabetes: A Randomized Clinical Trial. *Jama* **2017**, *318*, 1460-1470, doi:10.1001/jama.2017.14752.
14. Aroda, V.R.; Rosenstock, J.; Terauchi, Y.; Altuntas, Y.; Lalic, N.M.; Morales Villegas, E.C.; Jeppesen, O.K.; Christiansen, E.; Hertz, C.L.; Haluzík, M. PIONEER 1: Randomized Clinical Trial of the Efficacy and Safety of Oral Semaglutide Monotherapy in Comparison With Placebo in Patients With Type 2 Diabetes. *Diabetes Care* **2019**, *42*, 1724-1732, doi:10.2337/dc19-0749.

15. Rodbard, H.W.; Rosenstock, J.; Canani, L.H.; Deerochanawong, C.; Gumprecht, J.; Lindberg, S.; Lingvay, I.; Søndergaard, A.L.; Treppendahl, M.B.; Montanya, E. Oral Semaglutide Versus Empagliflozin in Patients With Type 2 Diabetes Uncontrolled on Metformin: The PIONEER 2 Trial. *Diabetes Care* **2019**, *42*, 2272-2281, doi:10.2337/dc19-0883.
16. Mosenzon, O.; Blicher, T.M.; Rosenlund, S.; Eriksson, J.W.; Heller, S.; Hels, O.H.; Pratley, R.; Sathyapalan, T.; Desouza, C. Efficacy and safety of oral semaglutide in patients with type 2 diabetes and moderate renal impairment (PIONEER 5): a placebo-controlled, randomised, phase 3a trial. *Lancet Diabetes Endocrinol* **2019**, *7*, 515-527, doi:10.1016/s2213-8587(19)30192-5.
17. Bækdal, T.A.; Breitschaft, A.; Navarria, A.; Hansen, C.W. A randomized study investigating the effect of omeprazole on the pharmacokinetics of oral semaglutide. *Expert Opin Drug Metab Toxicol* **2018**, *14*, 869-877, doi:10.1080/17425255.2018.1488965.
18. Bækdal, T.A.; Borregaard, J.; Hansen, C.W.; Thomsen, M.; Anderson, T.W. Effect of Oral Semaglutide on the Pharmacokinetics of Lisinopril, Warfarin, Digoxin, and Metformin in Healthy Subjects. *Clin Pharmacokinet* **2019**, *58*, 1193-1203, doi:10.1007/s40262-019-00756-2.
19. Jordy, A.B.; Albayaty, M.; Breitschaft, A.; Anderson, T.W.; Christiansen, E.; Houshmand-Øregaard, A.; Manigandan, E.; Bækdal, T.A. Effect of Oral Semaglutide on the Pharmacokinetics of Levonorgestrel and Ethinylestradiol in Healthy Postmenopausal Women and Furosemide and Rosuvastatin in Healthy Subjects. *Clin Pharmacokinet* **2021**, *60*, 1171-1185, doi:10.1007/s40262-020-00976-x.
20. Granhall, C.; Bækdal, T.A.; Breitschaft, A.; Søndergaard, F.L.; Anderson, T.W.; Thomsen, M. Absence of QTc Prolongation with Sodium N-(8-[2-Hydroxybenzoyl] Amino) Caprylate (SNAC), an Absorption Enhancer Co-Formulated with the GLP-1 Analogue Semaglutide for Oral Administration. *Diabetes Ther* **2021**, *12*, 2599-2610, doi:10.1007/s13300-021-01106-x.
21. Hauge, C.; Breitschaft, A.; Hartoft-Nielsen, M.L.; Jensen, S.; Bækdal, T.A. Effect of oral semaglutide on the pharmacokinetics of thyroxine after dosing of levothyroxine and the influence of co-administered tablets on the pharmacokinetics of oral semaglutide in healthy subjects: an open-label, one-sequence crossover, single-center, multiple-dose, two-part trial. *Expert Opin Drug Metab Toxicol* **2021**, *17*, 1139-1148, doi:10.1080/17425255.2021.1955856.
